# Conjugate Heat Transfer Investigation on Swirl-Film Cooling at the Leading Edge of a Gas Turbine Vane

**DOI:** 10.3390/e21101007

**Published:** 2019-10-15

**Authors:** Haifen Du, Ziyue Mei, Jiayao Zou, Wei Jiang, Danmei Xie

**Affiliations:** Key Laboratory of Hydraulic Machinery Transients (MOE), School of Power and Mechanical Engineering, Wuhan University, Wuhan 430072, China; du_haifen@whu.edu.cn (H.D.); zymei@whu.edu.cn (Z.M.); 2019282080095@whu.edu.cn (J.Z.); jiangwei@whu.edu.cn (W.J.)

**Keywords:** film cooling, swirl cooling, overall effectiveness, heat transfer, pressure loss

## Abstract

Numerical calculation of conjugate heat transfer was carried out to study the effect of combined film and swirl cooling at the leading edge of a gas turbine vane with a cooling chamber inside. Two cooling chambers (C_1_ and C_2_ cases) were specially designed to generate swirl in the chamber, which could enhance overall cooling effectiveness at the leading edge. A simple cooling chamber (C_0_ case) was designed as a baseline. The effects of different cooling chambers were studied. Compared with the C_0_ case, the cooling chamber in the C_1_ case consists of a front cavity and a back cavity and two cavities are connected by a passage on the pressure side to improve the overall cooling effectiveness of the vane. The area-averaged overall cooling effectiveness of the leading edge ($\bar{\bar{\phi }}$) was improved by approximately 57%. Based on the C_1_ case, the passage along the vane was divided into nine segments in the C_2_ case to enhance the cooling effectiveness at the leading edge, and $\bar{\bar{\phi }}$ was enhanced by 75% compared with that in the C_0_ case. Additionally, the cooling efficiency on the pressure side was improved significantly by using swirl-cooling chambers. Pressure loss in the C_2_ and C_1_ cases was larger than that in the C_0_ case.

## 1. Introduction

Gas turbines are widely used in aero-propulsion system, ship power, and industrial power generation. The inlet temperature of modern gas turbines exceeds 2000 K, which is far beyond the melting points of component materials. Thermal degradation of turbine parts has the potential to cause major engine problems, giving rise to costly repairs and downtime [1,2,3]. The blade leading edge bears a higher heat load and requires a stricter cooling method because of the direct scour of hot gas. An effective cooling technique is one of the most important parts of the thermal design. Various cooling methods have been investigated for many years. Han [4] presented the research activities in gas turbine blade cooling. The cooling methods used for the blade/vane are divided into in two categories: external film cooling and internal cooling [5].

Many researchers have devoted themselves to studying film cooling or internal convective cooling technology. Several film cooling methods have been studied and are used in many advanced engineering applications [6]. In film cooling, cool air is discharged from rows of holes on the vane surface, and the injected air forms a thin film on the surface acting as a buffer between the hot gas and the vane [7]. Because of the high importance and widespread application of film cooling, research into its various aspects has seen a tremendous increase in the last 10–15 years. The publications relating directly or indirectly to film cooling are too numerous to recount here, and they studied the effects of film hole internal fluid dynamics, interactions with the mainstream gas flow, vortex production, hole shaping, orientation, spacing, hole length-to-diameter ratio, density ratio and blowing strength, momentum flux ratio, mainstream turbulence intensity and so on [8,9,10,11,12,13,14,15]. A series of internal cooling methods such as impingement cooling have been used in the turbine vane leading edge. However, impingement cooling cannot lead to a uniform temperature distribution in the blade, and an excessive film cooling flow rate can adversely affect the mainstream aerodynamic effectiveness. To overcome the difficulties above, new concepts of cooling technologies for the leading edge need to be developed and investigated carefully. Swirl cooling is a new but efficient method with high heat transfer intensity and low flow losses. Hay and West [16] initially proposed that swirl chambers can be introduced to the internal cooling of turbine. Their research demonstrated that the heat transfer augmentation factor of fully developed axial turbulent flow is approximately eight times that near the inlet region. Seeking further improvements in the swirl cooling performance, some researchers [17,18,19,20,21] investigated the effects of Reynolds number, swirl intensity, and geometrical parameters on swirl cooling. However, the majority of previous research into swirl cooling depended on simple pipe models with inappropriate sizes, which were not consistent with cooling structures in a gas turbine vane [22,23].

Most of them focus on the gas turbine components cooled by only film cooling or internal cooling, not overall cooling, which is more complicated. However, heat transfer performance of a vane cooled by overall cooling (both external cooling and internal cooling) is less studied. In terms of the composite cooling method that has been studied, impingement cooling coupled with film cooling is the most popular cooling method. Some studies incorporating impingement cooling and film cooling were performed in different areas of a C3X vane [24,25,26,27]. Few studies have been conducted on overall cooling incorporating film cooling and swirl cooling. Although the authors of [28,29] investigated the cooling performance of a turbine blade leading edge with a simplified three-dimensional vortex chamber structure and film holes, their work focused on swirl cooling effectiveness and adiabatic film cooling effectiveness, not overall cooling. Conjugate heat transfer models predict a significant difference in temperature predictions in comparison with the adiabatic models, because of the importance of considering the heat conduction in the metal to accurately predict surface temperature [30]. Therefore, conjugate heat transfer models accurately predict overall cooling performance of gas turbine vane. However, almost no work was done to use conjugate heat transfer models to investigate the overall cooling effectiveness of film-swirl cooling for a gas turbine vane. 

In the current work, a numerical calculation of conjugate heat transfer (CHT) was carried out to study the effect of overall cooling incorporating film and swirl cooling at the leading edge of a gas turbine vane on the flow structure and heat transfer. Three kinds of cooling chambers were designed, among which two kinds of cooling chambers were designed to generate swirl flow. The influence of different cooling chambers on cooling performance was investigated to provide the basic idea reference for swirl structure design in film-swirl cooling. The pressure loss due to designed cooling chambers was studied.

## 2. Numerical method

### 2.1. Geometrical Details

**Figure 1:**
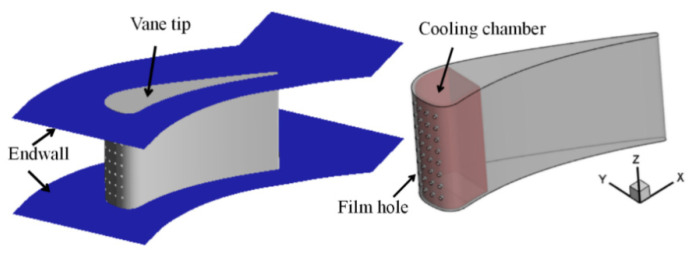
Geometry of the turbine vane with a cooling chamber.

To investigate the cooling performance of film-swirl cooling on a turbine vane, a geometrical model was established based on a vane. As illustrated in Figure 1, four rows of film holes are located at the leading edge of the vane with a cooling chamber inside. The coolant flows into the cooling chamber from the coolant inlet and flows out of the vane through the film holes.

**Figure 2:**
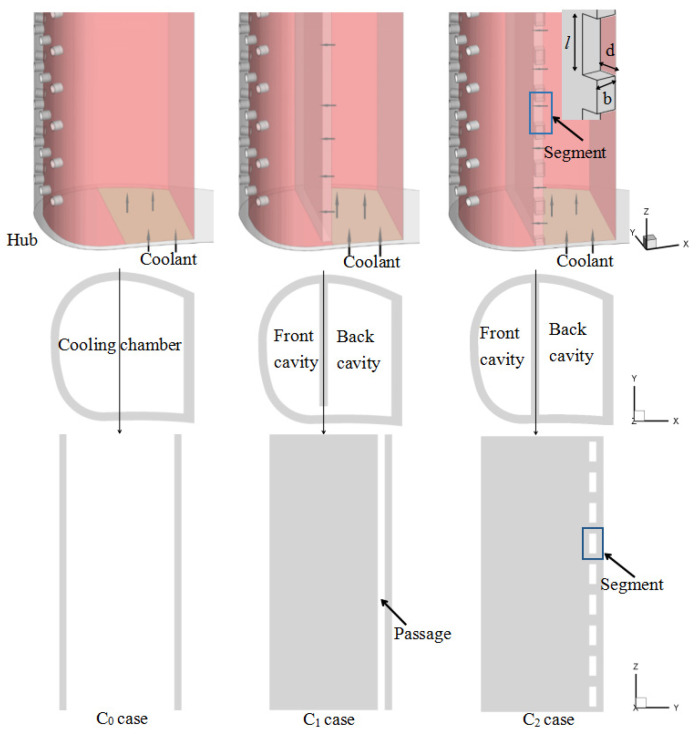
Different cooling chamber configurations.

Three different configurations of cooling chambers were used for comparison, and the differences are presented in Figure 2. The cooling chamber is composed of a front cavity and a back cavity, which are connected by a passage (in C_1_ and C_2_ cases). In the C_2_ case, the passage along the vane is divided into nine segments, and the position of the segment is staggered with positions of the film holes. Table 1 lists the detailed geometrical parameters.

### 2.2. Computational Grids

**Figure 3:**
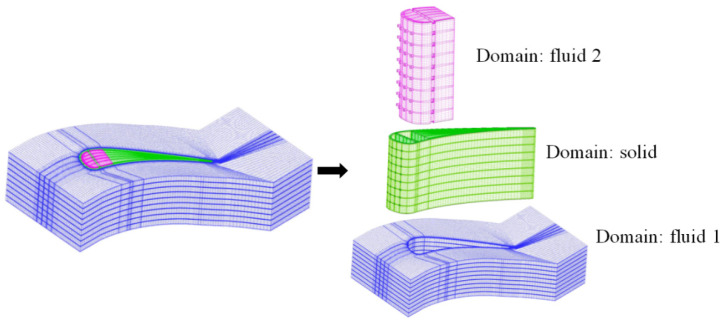
Grid in the computational model.

The grid in the C_2_ CHT model is presented in Figure 3. Three blocks were used to distribute the grids throughout the computational domain: mainstream region (Fluid 1), vane region (solid), and coolant region (fluid 2). As the grid resolution increases, more scales are resolved by the grids, leading to better and more accurate predictions. Therefore, prior to the actual numerical simulation, a grid independence study for the conditions of the C_2_ CHT model at MFR = 0.75% was performed by using four different grid arrangements with 1,931,191; 2,501,791; 3,112,664 and 3,628,721 cells, as presented in Table 2.

**Figure 4:**
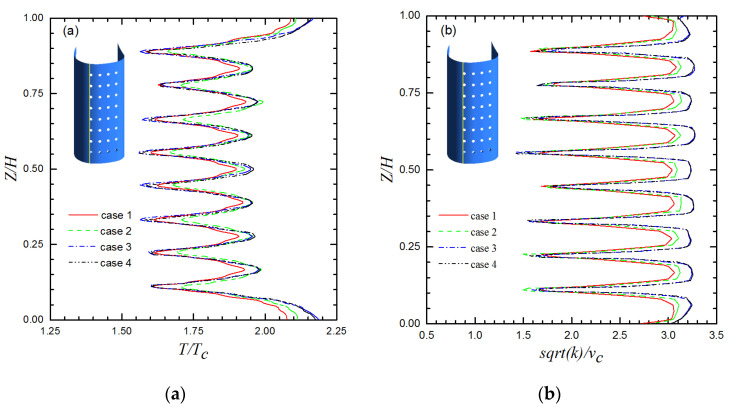
Grid independence study using temperature and turbulent kinetic energy along the span direction downstream of Row 4. (**a**) temperature along the span direction downstream of Row 4; (**b**) turbulent kinetic energy along the span direction downstream of Row 4.

The comparisons were made using temperature and turbulent kinetic energy along the span direction downstream of the Row 4 film holes, as illustrated in Figure 4. The height of the first mesh layer is 1 × 10^−6^ m, and the growth factor for the cells is 1.1. We encrypted the grid near the wall so that the y^+^ value of the wall was less than 3. The numerical results indicate that the variation of the variables for judgment changes by less than 1% when the number of meshes increases from 3.1 million to 3.6 million. Based on these comparisons, the Case 3 grid was selected.

### 2.3. Validation and Boundary Conditions

ANSYS FLUENT was chosen as the computational fluid dynamic tool by considering the fluid–structure interaction. A three-dimensional pressure-based compressible flow solver was used to solve the N-S equation. The conservation laws for the current study are as follows:

Continuity equation:

(1) $$\frac{\partial \rho }{\partial t}+\nabla \cdot \left(\rho \vec{U}\right)=0$$

Momentum equation:

(2) $$\frac{\partial \left(\rho \vec{U}\right)}{\partial t}+\nabla \cdot \left(\rho \vec{U}\cdot \vec{U}\right)=-\nabla p+\nabla \cdot \tau +S_{M}$$

(3) $$\tau =\mu \left(\nabla \vec{U}+{\left(\nabla \vec{U}\right)}^{T}\right)-\frac{2}{3}\nabla \cdot \vec{U}I$$

Energy equation:

(4) $$\frac{\partial \left(\rho E\right)}{\partial t}+\nabla \cdot \left(\vec{U}\left(\rho E+p\right)\right)=\nabla \cdot \left(k_{eff}\nabla T-\sum_{j}h_{j}{\vec{J}}_{j}+\left(\tau \cdot \vec{U}\right)\right)+S_{h}$$

where $\vec{U}$ is the velocity vector, *p* is the static pressure, *τ* is the stress tensor, *S_M_* is the momentum source, *I* is the unit tensor, *E* is the total energy, $k_{eff}$ is the effective conductivity, *h* is the sensible enthalpy, ${\vec{J}}_{j}$ is the diffusion flux of species *j*, and $S_{h}$ is the energy source.

**Figure 5:**
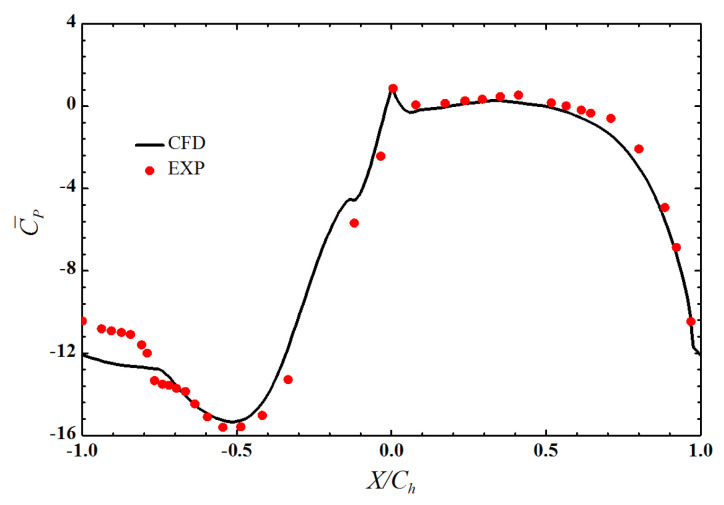
Comparison of spanwise averaged coefficient of pressure.

The κ−ω SST model was adopted as the turbulence model in this study. The computational model was validated by comparing the spanwise averaged coefficient of pressure distribution on the vane obtained computationally with the experiment carried out by Chandran and Prasad [31]. Figure 5 indicates that a good correspondence exists between the experimental and computational results, thereby validating the computational methodology, including the mesh and the turbulence model adopted for the computations.

The main stream inlet was set as the pressure inlet; the total pressure was 15 bar; the temperature was 1962 K; and the turbulence intensity was 5%. The outlet was defined as the pressure outlet, and the pressure was 9 bar. The coolant inlet was set as mass flow inlet based on MFR = 0.75%; the total pressure was 15 bar; the temperature was 600 K; and the turbulence intensity was 5%. The mass flow rate ratio MFR ranged from 0.25% to 1.00%. The vane material was 310 stainless steel. The material density was 8030 kg/m^3^; the specific heat was 502 *J/(kg∙K)*; and the thermal coefficient *K_s_* was fitted by the temperature polynomial as $K_{s}=9.9105+0.0115T$. The wall boundary with no slip condition was utilized to calculate overall cooling effectiveness. The endwall, vane tip and hub were adiabatic walls without thickness. The dynamic viscosity *µ* and the thermal coefficient *K* of the gas were expressed with Sutherland formulae as functions of temperature:

(5) μ∞(T)=μ0(TT0)32T0+ST+S

(6) K∞(T)=λ0(TT0)32T0+ST+S

where $\mu_{0}=1.7894\times 10^{-5}Pa\cdot s,$ $T_{0}=273.11K,$ S=110.56K, and $\lambda_{0}=0.0261W/(m\cdot s).$ The specific heat capacity $C_{\infty }$ of the gas was fitted by the temperature polynomial as follows:

(7) $$C_{\infty }=a_{0}+a_{1}T+a_{2}T^{2}+a_{3}T^{3}+a_{4}T^{4}$$

where $a_{0}=957.110256$, $a_{1}=0.236523$, $a_{2}=5.141114\times 10^{-6}$, $a_{3}=-3.391745\times 10^{-9}$, and $a_{4}=-6.092965\times 10^{-12}$.

## 3. Results and Discussion

### 3.1. Flow Distribution

**Figure 6:**
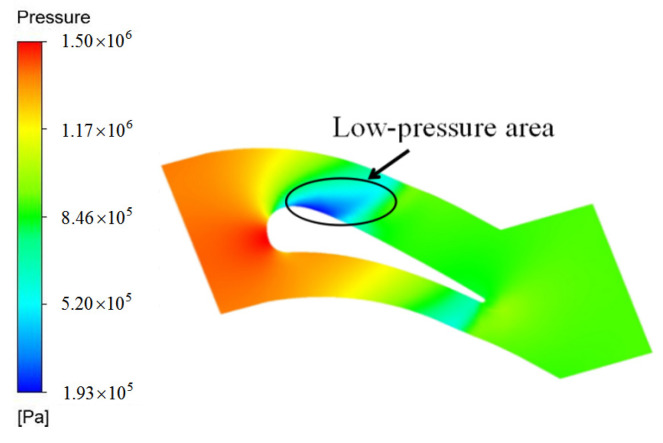
Gas static pressure in the C_1_ case at Z/H = 0.5.

The vane profile plays an important part in aerodynamic performance. The vane calculated in this work is a fore-loaded vane. The distribution of the mainstream flow field of the C_1_ case is reported as an example. As illustrated in Figure 6, a low-pressure region is located at the suction surface of the leading edge.

**Figure 7:**
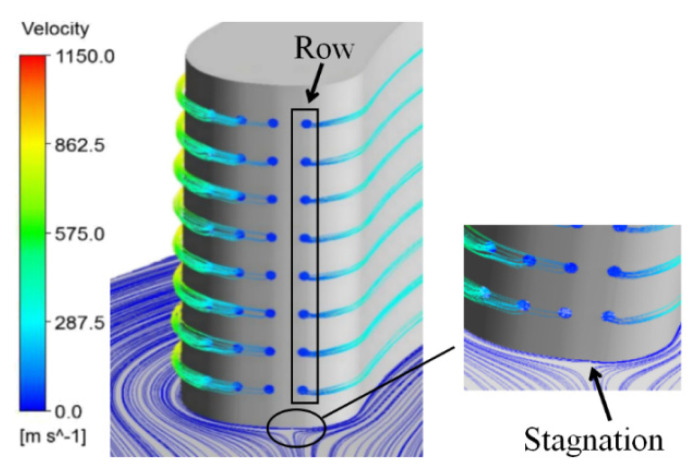
The streamlines from film holes and streamlines at the endwall.

The coolant velocity streamlines from the film holes and the streamlines at the endwall are illustrated in Figure 7. It can be observed that the coolant flows to the pressure side through Row 1, and the coolant flows to the suction side through the other rows of film holes. This phenomenon is caused by the stagnation point of the vane being located between Row 1 and the other rows. This is important for cooling performance.

The cooling effectiveness of the vane’s leading edge was mainly studied. Only the data on the leading edge (−16 ≤ *X/D* ≤ 16) were extracted for analysis, and a negative number “-” means the suction side here.

**Figure 8:**
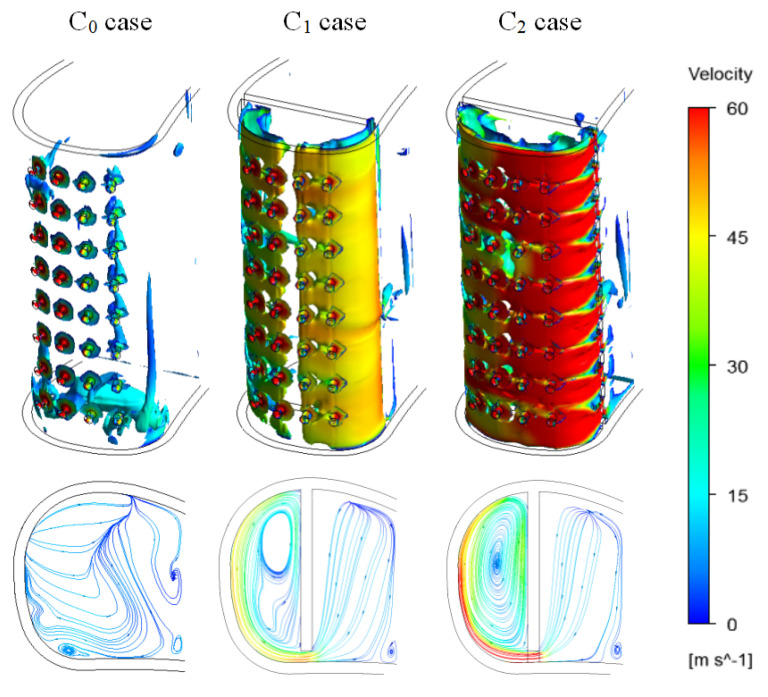
Vortex core region and streamlines in Fluid 2 at Z/H = 0.5.

Figure 8 presents the vortex core region and surface streamlines in the cooling chambers of the three cases. It indicates that two different types of cooling chamber configuration (C_1_ and C_2_) are selected to generate a swirling flow. In C_1_ and C_2_ cases, the velocity increases, and the swirling flow occurs in the front cavity because of the coolant flowing through the passage of the cooling chamber. This result indicates that the vane is cooled with swirling film cooling in C_1_ and C_2_ cases. In the two cases, flow velocity and vortex core region in the cooling chamber are both larger than C_0_ case. Additionally, flow velocity in the C_2_ case’s front cavity is higher, and the vortex core region is larger than those in the C_1_ front cavity. 

**Figure 9:**
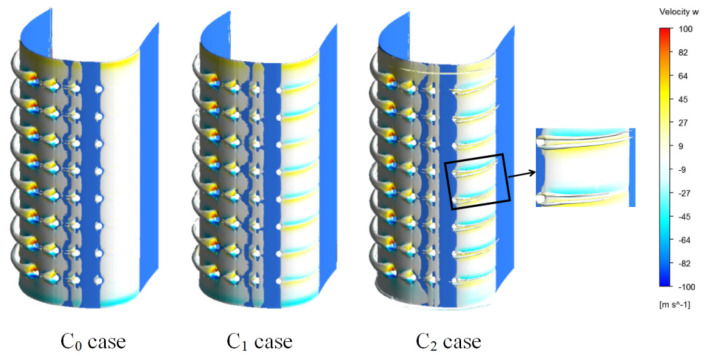
Vortex core region (swirling strength level = 0.025) and velocity w.

The relative coolant mass flow rate at the film holes exit in each row is given in Table 3. In the C_0_ case, gas flows into the coolant chamber through Row 1. Therefore, in the C_0_ case, the mixture of gas and coolant results in a swirl flow in the coolant chamber (Figure 8). As shown in Figure 9, the coolant flows out of the vane with the appearance of a counter-rotating vortex pair downstream of the film holes. Meanwhile, Figure 9 illustrates that no coolant flows out the vane through Row 1 in the C_0_ case. In three cases, one can note that the sense of rotation of the vortex pair from the holes goes outward; this generally improves the lateral coverage of coolant at downstream of the holes. The larger lateral coverage of coolant contributes to larger film cooling effectiveness. It means that the structure of cooling chamber (internal cooling) has influence on film cooling. In the C_1_ and C_2_ cases (the internal cooling is swirl cooling), coolant mass flow and vortex core region on the pressure side are larger than C_0_ case. Compared to that in the C_1_ case, the lateral coverage of coolant on the pressure side is larger in the C_2_ case.

### 3.2. Heat Transfer and Pressure Loss

The wall heat flux q at the heat transfer coefficient wall is calculated by the following equation:

(8) $$q=h_{0}(T_{0}-T_{w})$$

where $h_{0}$ is the external heat transfer coefficient. $T_{0}$ is the external boundary temperature, which is the temperature of the fluid near the wall in this paper, that is, the wall temperature. This parameter means that the wall is cooled by flow at q<0 and heated by flow at q>0. $q_{ref}$ is the reference value of the wall heat flux.

**Figure 10:**
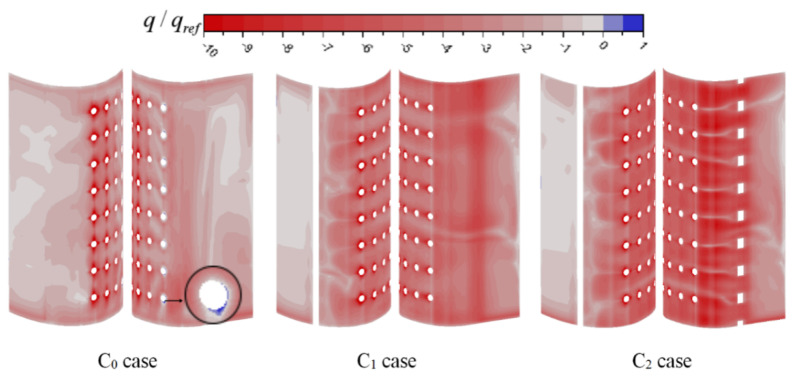
Wall heat flux on the inside wall of the vane.

The distribution of the wall heat flux on the inside wall of the vane is presented in Figure 10. The red region (q>0) is the region of the vane cooled by the coolant, and the blue region (q>0) is the region heated by gas flowing into the coolant chamber through film holes. In the C_0_ case, one can note that the region near the Row 1 film holes is heated by gas. As stated above, gas flows into the chamber through Row 1 film holes in the C_0_ case. The larger absolute value of the negative wall heat flux means that the coolant carries more heat from the wall, and the internal cooling efficiency is larger. Compared with that in the C_0_ case, the internal cooling efficiency in the C_1_ and C_2_ cases is larger, because of the larger velocity and swirl. It also proves that swirl cooling brings higher internal cooling efficiency. Meanwhile, the internal cooling efficiency in the C_2_ case is the largest, especially on the pressure side. This is due to the velocity in the passage in C_2_ case is the largest.

**Figure 11:**
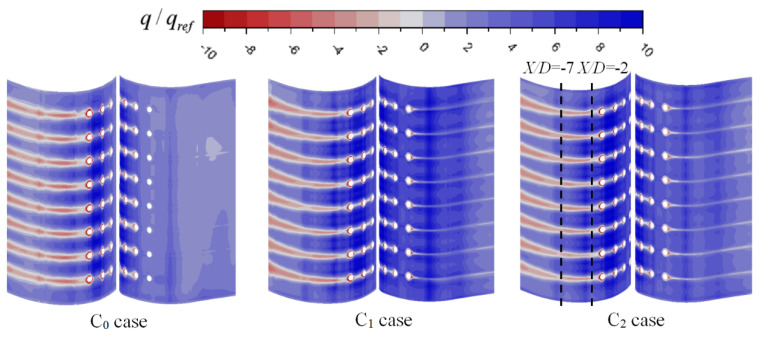
Wall heat flux on the outside wall of the vane.

Figure 11 shows the contours of the wall heat flux on the outside wall of the vane. The red region is cooled by the coolant from film holes, and the negative wall heat flux represents the film cooling effect. In addition, no coolant covers the blue region. For three cases, the film cooling efficiency at the exit of film holes is significant, except for Row 1 in the C_0_ case. The film cooling efficiency at downstream of the film holes is high. It is because that the coolant covers only the region downstream of the film holes and mainstream gas directly in contact with other areas of the vane. Along the streamline direction downstream of the Row 4 film holes, the film cooling efficiency first decreases and then increases. This is because the coolant from the film holes is first lifted off the vane and adheres to the vane afterwards. On the pressure side of the C_1_ and C_2_ cases, the region downstream of the Row 1 film holes is cooled by film cooling by coolant from the film holes. Therefore, film cooling efficiency on the pressure side in the two cases is larger than C_0_ case. In other areas, except for downstream of the film holes, wall heat flux is the largest in the C_2_ case and the smallest in the C_0_ case. As demonstrated previously, compared with C_1_ case, the internal cooling effectiveness in the C_2_ case is higher than C_1_, making the coolant temperature higher and temperature vane lower. Therefore, in the C_2_ case, heat flux between gas and the vane is larger than C_1_ case.

**Figure 12:**
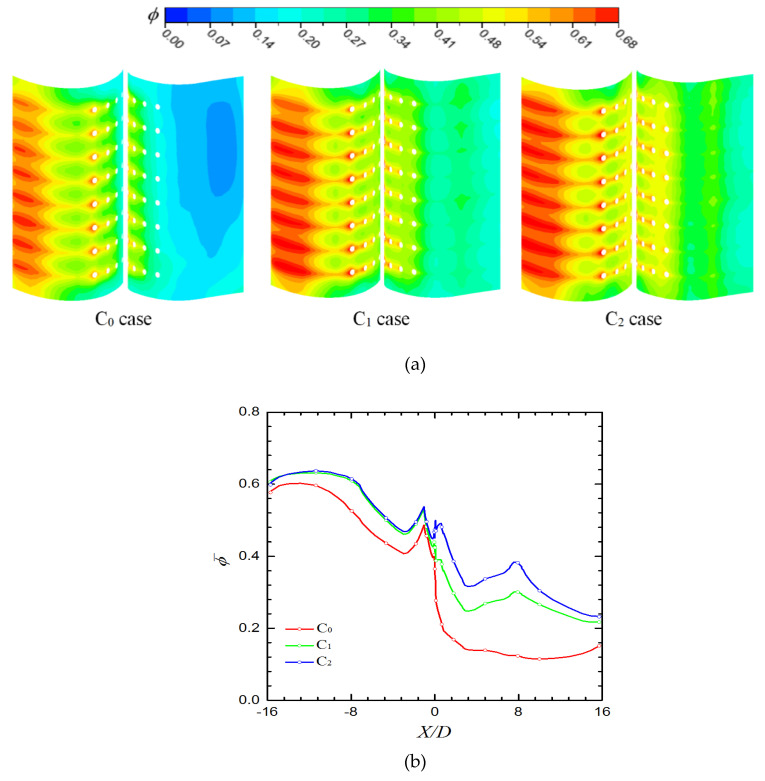
Overall cooling effectiveness of the three cases. (**a**) overall cooling effectiveness on the outside wall of the vane; (**b**) laterally averaged overall cooling effectiveness.

In Figure 12a, for the three cases, ϕ at the exit downstream and that of film holes is large. In addition to internal cooling, the film cooling effectiveness is satisfactory at the holes exits. Thus, the overall cooling effectiveness is larger in these region than other regions. The highest cooling effectiveness appears in the region (−15 < *X/D* < −7) due to the low-pressure area depicted in Figure 6. Compared with the C_0_ case, ϕ on the pressure side is high in the C_1_ and C_2_ cases, and a high ϕ area is in the region (7 < *X/D* < 8), which corresponds to the passage. As shown in Figure 12b, the laterally averaged overall cooling effectiveness in the C_1_ and C_2_ cases is larger than that in the C_0_ case. In particular, no coolant flows through Row 1 of the C_0_ case. This means that configurations of the C_1_ and C_2_ cases improve mass flow of the coolant through film holes on the pressure side. On the pressure side, overall cooling effectiveness of the C_2_ case is the highest because of significant internal and film cooling effect, which is followed by that of the C_1_ case. Compared with the C_0_ case, $\bar{\phi }$ is improved by 215% and 147% in C_2_ and C_1_ cases, respectively, at *X/D* = 7.6. In this area, the internal cooling effectiveness is high because of the large velocity of the coolant. In the region (−7 < *X/D* < −2.5), compared with the C_0_ case, in the C_1_ and C_2_ cases, internal cooling effectiveness is higher and film cooling effectiveness is lower in this region. Moreover, the overall cooling effectiveness in the C_2_ case is higher than the other two cases. This result reveals that the effect of internal cooling on overall effectiveness is significant in this region.

The pressure loss coefficient $\xi_{p}$ is calculated by following equation:

(9) $$\xi_{p}=\frac{\frac{m_{\infty }}{m_{\infty }+m_{c}}P_{t,\infty }+\frac{m_{c}}{m_{\infty }+m_{c}}P_{t,c}-P_{t,out}}{\frac{m_{\infty }}{m_{\infty }+m_{c}}P_{t,\infty }+\frac{m_{c}}{m_{\infty }+m_{c}}P_{t,c}}$$

where $P_{t,\infty }$ is gas inlet total pressure, $P_{t,c}$ is coolant inlet total pressure, and $P_{t,out}$ is vane outlet total pressure.

**Figure 13:**
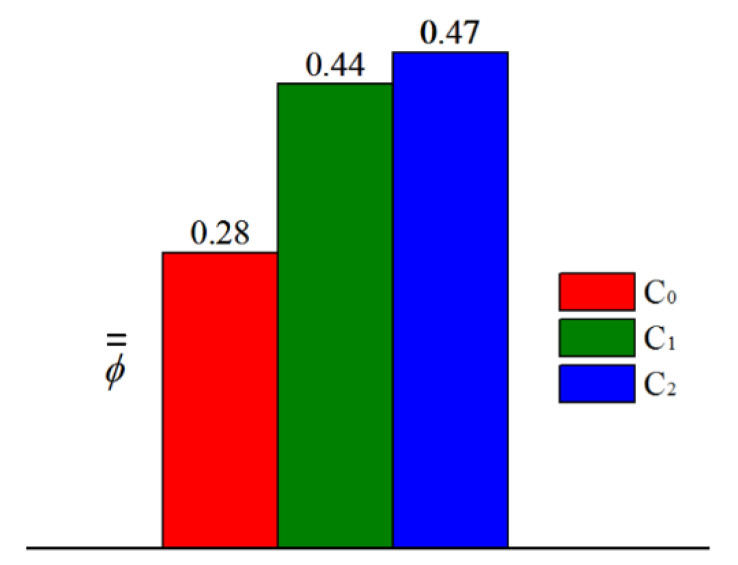
Area-averaged overall cooling effectiveness in different cases.

**Figure 14:**
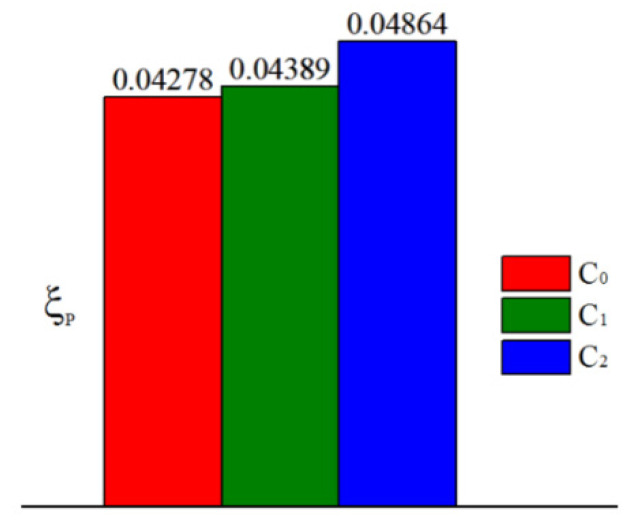
Pressure loss coefficient in different cases.

Figure 13 presents area-averaged overall cooling effectiveness (−16 ≤ *X/D* ≤ 16) in the three cases. The area-averaged overall cooling effectiveness $\bar{\bar{\phi }}$ is enhanced by approximately 75% and 57% in the C_2_ and C_1_ cases, respectively, compared with that in the C_0_ case. This means that swirl-film cooling works well in cooling the leading edge of the vane. However, pressure loss in the C_1_ and C_2_ cases is larger than that in the C_0_ case (as illustrated in Figure 14). Particularly in the C_2_ case, pressure loss coefficient $\xi_{p}$ increases by 13.7% compared with that in the C_0_ case; $\xi_{p}$ in the C_1_ case increases only by 2.6%.

## 4. Conclusions

We investigated the overall cooling effectiveness of film and swirl cooling at the leading edge of a gas turbine vane. Three cases were evaluated to study overall cooling incorporating film and internal cooling. In the C_1_ and C_2_ cases, the internal cooling is swirl cooling. Two different types of cooling chambers (C_1_ and C_2_ cases) were designed to develop swirling flow. Compared with the C_0_ case, internal cooling effectiveness is enhanced by using cooling chambers of the C_1_ and C_2_ cases, and film cooling effectiveness on the pressure side was improved. It means that swirl cooling improves internal cooling effectiveness. Film cooling efficiency is affected by internal cooling method. Therefore, calculation of conjugate heat transfer is necessary to study overall cooling in the gas turbine vane. The results indicate that the effect of swirl cooling on overall effectiveness is significant. The overall cooling effectiveness ϕ in the C_1_ and C_2_ cases is larger than that in the C_0_ case. The area-averaged overall effectiveness of the leading edge is enhanced by approximately 75% and 57% in the C_2_ and C_1_ cases, respectively. The designed cooling chamber in the C_2_ case is beneficial for improving cooling efficiency. Overall cooling effectiveness of the leading edge can be improved by optimizing the cooling chamber. Pressure loss in the C_2_ and C_1_ cases is larger than that in the C_0_ case. The pressure loss increases with the increase of cooling performance. The pressure loss coefficient $\xi_{P}$ is increased by 13.7% in the C_2_ case and by 2.6% in the C_1_ case compared with that in the C_0_ case. 

## Figures and Tables

**Table 1:** Geometric details of the vane.

Parameter	Values (mm)	Parameter	Values (mm)
*H*	76.2	*b*	2
*L*	117.73	*d*	2
*D*	2	*l*	5.47

**Table 2:** Grid arrangements for the computational domain.

Grid Case	Fluid 1	Solid	Fluid 2	Total Number of Grids
1	1,048,858	595,201	287,132	1,931,191
2	1,393,573	742,495	365,723	2,501,791
3	1,708,962	953,661	450,041	3,112,664
4	1,897,700	1,184,440	546,581	3,628,721

**Table 3:** Relative coolant mass flow rate at film hole exit *m/m_c_%.*

	C_0_	C_1_	C_2_	
Row 1	--	10.26	12.66	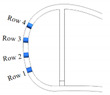
Row 2	8.71	5.74	6.26	
Row 3	31.96	25.81	25.16	
Row 4	59.33	58.19	55.92	

“-” indicates flow ingestion into the vane.

## Nomenclature

*H*	Vane height (m)
*C_h_*	Chord length (m)
*D*	Diameter of the film hole (m)
*b*	Passage width (m)
*d*	Passage segment length (m)
*l*	Passage segment height (m)
*C_P_*	Coefficient of pressure, $\left(p-p_{\infty })/(0.5\times \rho \times \nu_{\infty }^{2}\right)$
$\bar{C_{p}}$	Spanwise averaged coefficient of pressure
*K*	Thermal coefficient (W/m^2^ K)
*U*	Velocity [m/s]
*C*	Specific heat capacity (J/(kg·K))
*T*	Temperature (K)
*P*	Pressure (Pa)
*m*	Mass flow (kg/s)
*q*	Wall heat flux (W/m^2^)
*h*	Heat transfer coefficient (-)
*MFR*	Mass flow rate, $m_{c}/m_{\infty }$ (-)
*Re*	Reynolds number (-)
*M*	Blowing ratio, $\left(\rho_{c}U_{c})/(\rho_{\infty }U_{\infty }\right)$ (-)
*Z*	The coordinate in the vane height direction (m)
*X*	The coordinate in the streamwise direction (m)
**Greek Symbols**	
*μ*	Dynamic viscosity (N·s/m^2^)
*ρ*	Density (kg/m^3^)
*ϕ*	Overall cooling effectiveness, $\left(T_{\infty }-T_{w})/(T_{\infty }-T_{c}\right)$ (-)
$\bar{\phi }$	Averaged overall cooling effectiveness (-)
$\bar{\bar{\phi }}$	Area-averaged overall cooling effectiveness (-)
$\xi_{p}$	Pressure loss coefficient (-)
**Subscripts**	
w	Wall
c	Inlet coolant
∞	Mainstream
s	Solid
